# Direct probing of contact electrification by using optical second harmonic generation technique

**DOI:** 10.1038/srep13019

**Published:** 2015-08-14

**Authors:** Xiangyu Chen, Dai Taguchi, Takaaki Manaka, Mitsumasa Iwamoto, Zhong Lin Wang

**Affiliations:** 1 Beijing Institute of Nanoenergy and Nanosystems, Chinese Academy of Sciences, Beijing 100083, China; 2 Department of Physical Electronics, Tokyo Institute of Technology, 2-12-1 S3-33 O-okayama, Meguro-ku Tokyo 152-8552 Japan; 3 School of Materials Science and Engineering, Georgia Institute of Technology, Atlanta, Georgia 30332-0245, USA

## Abstract

Contact electrification between two different materials is one of the oldest fields of study in solid-state physics. Here, we introduced an innovative system based on optical electric-field-induced second harmonic generation (EFI-SHG) technique that can directly monitor the dynamic performance of the contact electrification on the surface of polyimide film. After the contact, the EFI-SHG system visualized briefly three relaxations of the tribo-induced charges on the surface of a polyimide film, a fast relaxation within 3 min followed by two much slower relaxations, which were possibly related to different charge diffusion routes. The contact electrification under several special experimental conditions (wind, water and steam) was studied to demonstrate the high flexibility and material selectivity of the EFI-SHG. The EFI-SHG studies confirmed the motion of the water can remove the surface charge, while the appearance and the evaporation of a thin water layer cannot enhance the charge diffusion. We anticipate that this experimental technique will find a variety of applications in the field of contact electrification and the development of the recently invented triboelectric nano generator.

The contact and the separation between two different materials lead to the charge transfer between two surfaces and this is so-called contact electrification or triboelectricity effect, which has been one of the oldest study fields for hundreds of years^1^,^2^. Though, this phenomenon has given birth to a lot of useful technologies, its physical origin has not been well identified^3^,^4^. In the last 4 years, a new type of energy harvesting technology based on this phenomenon, triboelectric nanogenerator (TENG), has also demonstrated a wide range of applications as a power source and self-powered sensors^5^,^6^,^7^,^8^. All these applications and developments have made it more necessary to achieve a better understanding of the dynamic performance of contact electrification. Electronic excessive charge transfer upon contact gives electrification, while orientational ordering of permanent dipoles also gives rise to generation of surface potentials. Accordingly, various tools and experimental methods have been proposed and designed for characterizing the electrification process^9^,^10^,^11^, for example, the scanning Kelvin probe microscopy (KPM) in several recent studies^4^,^9^. The common concept of these methods is based on the probing the electrical flux originating from electrified surface with an aid of electrical detectors. As a result, it is hard to discuss the physical origins of tribo-electricity, because electronic charges and electric dipoles produce electric flux toward the electrical detectors in the same way, according to the electro-magnetic theory of the Gauss law. Furthermore, surface electrostatic field is very easily disturbed by the electromagnetic and acoustic interference from environment. Thus, we must measure the electrification phenomena in a heavy vacuum chamber isolated from these noise, i.e., by using a Faraday cage^9^. Finally, the experimental reproducibility is usually challenged by the existence of dust particles, surface contaminants and so on^3^. It is therefore highly desirable to develop a technique that does not rely on electrical methods, but can selectively detect surface charges that originate from many physical origins.

A promising solution for completing these detailed studies of contact electrification is to develop an optical experimental method that allows electrostatic fields to be probed. In this paper, we demonstrate an innovative optical method for directly observing contact electrification and triboelectricity, which is an optical method based on optical second harmonic generation (SHG) techniques^12^,^13^. The molecular ordering by the tribo motion contributes to generation of non-zero SHG signal, while the static electric field in the targeted materials leads to the enhancement of optical electric-field-induced second harmonic generation (EFI-SHG)^12^,^13^. Choosing appropriate probing laser wavelength allows us to selectively activate SHG and EFI-SHG, and thus enables us to discuss the physical origin of the contact electrification. In the measurement, our targeted material was polyimide film, which is a well-studied electrical insulating material (low carrier density and high resistivity)^14^,^15^. Accordingly, the electric-field distribution probed by the EFI-SHG obeys the Laplacian field, which is the electric field in an insulator caused by charges induced on electrodes in the absence of any charges between the electrodes. Recent dynamics observation in organic electronics using the EFI-SHG^12^,^13^ has proved the capability of monitoring the generation and the relaxation of the electric field in organic semiconductor, where charges are transported inside the material. For the metal-polyimide contact’s case, surface electronic states in polyimide films is dominant within the region of 1 nm from the interface^14^,^15^ and thus the charges are statically distributed near the surface, which is different from semiconductor’s study^12^,^13^. The SHG response from polyimide film should also originate mostly from the first few nanometers region near the surface, which is similar to water-air interface’s case^16^,^17^. Therefore, the EFI-SHG system is quite suitable for studying the contact and tribo electrification. Meanwhile, the generation of the SHG signal has the material selectivity^18^ and we may selectively observe the SHG from only polyimide molecule in a complex system. Therefore, the EFI-SHG system is a novel approach for the study of contact electrification and triboelectricity effect. The optical SHG technique is also innovative in terms of probing contact electrification that arose from many possible sources, including environmental ones such as wind and water drops. This experimental flexibility also can allow us to monitor the internal dynamic performance of the electrification-based devices, such as TENG, in the real operation.

## Results

### Polyimide under AC voltage

**Figure 1:**
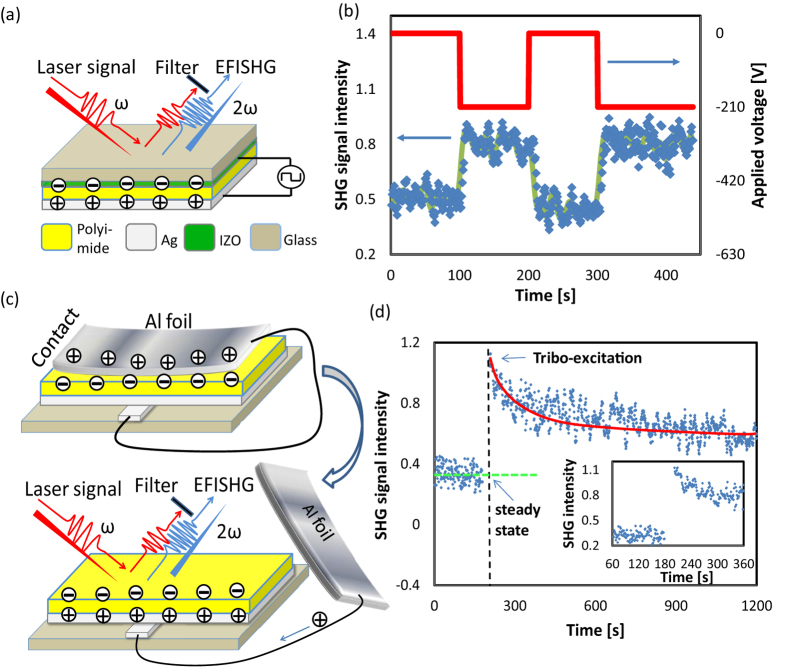
(**a**) The EFISHG experiment of polyimide film under applied power source (**b**) the observed SHG signal from the polyimide film with the external voltage, where the change of the SHG signal is caused by the EFI-SHG process. (**c**) The EFI-SHG experiment of polyimide film under tribo-excited electric field (**d**) the observed SHG signal from the polyimide film after directly contact with Al foil.

The polyimide sample with a Metal-Insulator-Metal (MIM) structure was prepared for studying the SHG generation under stable voltage source, as shown in Fig. 1(a). In the experiment, the polyimide (Kapton) film with a thickness of 50 μm was deposited on the IZO-glass substrate. Then, Ag electrode was deposited on the surface of the polyimide film to form a reflection surface for laser reflection. The Ag electrode was electrically grounded while the square AC voltage signal (0 V to −210 V) was applied on the IZO electrode, as displayed in Fig. 1(b). The changing of the SHG signal intensity from the sample (was related to EFI-SHG) was also illustrated in Fig. 1(b), where SHG signal shows a good agreement with the applied AC voltage signal. This experiment proved the possibility to generate EFI-SHG signal from thick polyimide film with the laser wavelength of 900 nm (SHG wavelength, 450 nm). It is important to note that the SHG signal can be also generated from Ag and IZO electrodes. However, this signal will not be changed with the applied voltage. The average visible light transmissivity of our IZO electrode is larger than 94%. Hence, we also neglected the influence from absorption and reflectivity of IZO layer in our experiment. Here, the SHG intensity was normalized by the maximum SH intensity at *t* = 0. Note that square-root of the SHG signal intensity is in proportion to the electric field strength^12^,^18^. From Fig. 1(b) we conclude that 0.1 square root of SHG signal (
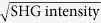
) corresponds to about 100 V voltage signal and thus the corresponding electric field is 2 × 10^6^ V/m. Hence, this relationship is available for the estimation of the tribo-generated electric field from the EFI-SHG observation.

### Polyimide under Tribo-excitation

We prepared a simple polyimide (Kapton) film as the tribo-material to work under the monitoring of the EFI-SHG system. The sample structure was shown in Fig. 1(c). The polyimide film with a thickness of 50 μm was placed on the top of Ag electrode and a piece of Al foil, which was electrically connected with Ag electrode, was used to contact with the top surface of the polyimide. According to previous study, polyimide surface will be negatively charged by the contact electrification process with Al foil^19^,^20^. After the separation, electrons stayed at the surface of the polyimide film while the holes transported from the Al foil to the Ag electrode on the bottom since the electrostatic charges stick to the shortest path^21^. Accordingly, a strong electric field was established across the polyimide film in the film thickness direction (which is enough for the generation of EFI-SHG) and laser signal continuously monitored the sample before and after the contact. One motion cycle for this measurement was defined as: Al foil firstly contacted with polyimide film, sled forward (or backward) for a small distance (3 ~ 5 mm) and then separated. In the experiments, some spongy cushion put behind the Al foil would enhance the contact and we verified that the results did not depend significantly on the duration time of contact or the pressure applied during contact (0.01 to 0.1 MPa), which is similar to the other researcher’s study^4^. The contact electrification process between polyimide film and other metals were also tested, as shown in Fig. S1. Here, we can confirm that the Al foil can offer us the highest surface charge density.

The SHG results from the tribo-excited polyimide film can be seen in Fig. 1(d), where the polyimide film experienced four motion cycles successively in order to reach its maximum surface charge density. In the experiment, we found that the SHG signal usually have a base line (≈0.3) in the steady state. As can be found in Fig. 1(d), after the tribo-excitation, the average intensity of SHG signal changed from 0.3 to 1.0, which indicates that the electric field changing across the polyimide film was Δ*E* ≈ 9 × 10^6^ V/m (450 V) and the related surface charge density changing was Δσ ≈ 318 μC/m^2^. (Here, the calculation is based on the normalized relationship between square root of SHG signal and local electric field obtained from Fig. 1(b) and the relative permittivity of polyimide is considered as 4.) The inset in the Fig. 1(d) was the enlarged figure of the SHG intensity changing from 60 second to 360 second. It can be seen that nearly 40% of tribo-induced charges diffused within three minutes (SHG signal changed from 1.0 to 0.7). However, the relaxation of the SHG signal from 0.7 to 0.5 experienced a rather long relaxation time, for about 15 minutes and from 0.5 to 0.3 would be even longer, for more than one hour. We have also examined the experiment with a break of the laser irradiation during the replication study of charge relaxation process. The observed results totally coincided with the results in Fig. 1(d). This confirmed that charge decay due to photoconductivity is negligibly small in the EFI-SHG measurement, since we chose probing laser wavelength in the near-infrared region where materials show almost no optical absorption and two-photon absorption of SHG can also reduce the photo-carrier generation to the negligible level^12^,^13^.

In order to verify the EFI-SHG measurements, we also performed Kelvin probe measurement for the tribo-excited polyimide film. The results can be found in Fig. S2 (a) and (b) in the supplementary information. The Kelvin probe measurement was performed in the vacuum condition to rule out the influences from the atmosphere. The Kelvin probe confirmed that the polyimide film was negatively charged through the contact with Al foil and the obtained surface potential from Kelvin probe was about −340 V, which was about 30% smaller than what we observed from the SHG signal (−450 V). The reason can be attributed to the relaxation of the surface charges during the setting of the probe. It is also interesting to note that the tribo-excited SHG signal always returns to a base line (0.3) in the steady state. If the charged polyimide film put in the open air for about 2 hours, the generated SHG signal will gradually return to this base line and after that the changing of the SHG signal will become very insignificant. However, this base line cannot be defined as the lowest surface potential. It is also quite possible that there will be some residual charges remained on the surface of polyimide to induce this steady signal and the relaxation of these residual charges may need an extremely long time. We will design some further experiments to confirm if this steady base line is the lowest surface potential state.

### Water application

**Figure 2:**
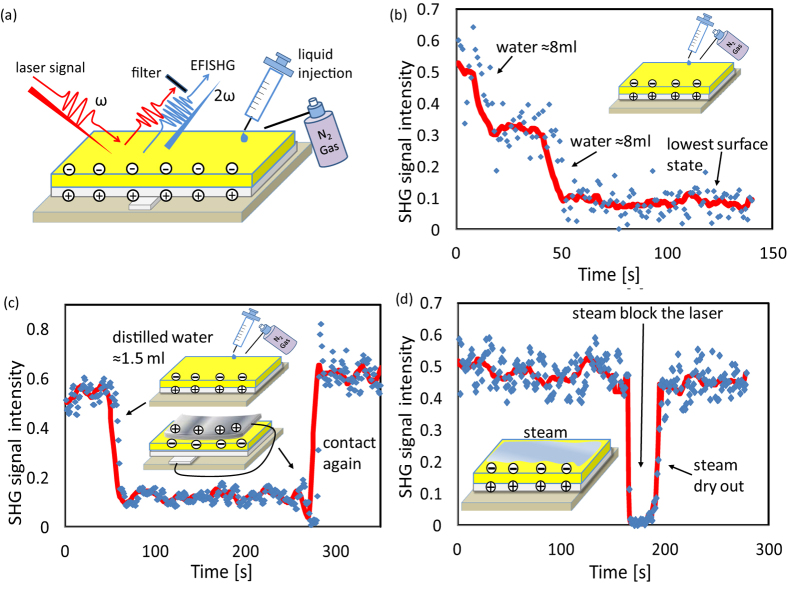
(**a**) The EFI-SHG experiment of polyimide film with water application. Here the N_2_ gas is always blowing the surface in order to remove the dropped water. Since the polyimide tape is hydrophobic, this gas blow is enough to clear the laser path (**b**) the observed SHG signal from the polyimide film with the water droplet. (**c**) The SHG signal from the polyimide film which firstly contacts with distilled water and then contacts with Al foil. (**d**) The SHG signal from the polyimide film that was covered by water steam. All the figures were drawn by X. C.

In order to determine the lowest surface potential state, we need to figure out some methods to remove the surface charge. It is well-known that the ion in the liquid water may neutralize the tribo-charges on the dielectric surface. Hence, we designed an experiment to observe the contact between water and polyimide. The experimental set up was shown in Fig. 2(a). The generation of SHG signal from water will be very weak at the laser wavelength of 900 nm^16^,^17^ and we only need to make sure that water will not block the laser path due to scattering. Hence, a N_2_ gas nozzle was placed near the surface of polyimide and the dry N_2_ kept blowing the surface in order to remove the water drop. Since the polyimide surface is hydrophobic, the blowing is enough to clear the laser path. It is important to note that the surface charge distribution will not be influenced by only blowing the surface with N_2_ gas, as can be seen in Fig. S3 in the supplementary information, where no significant changing happened with blowing the polyimide surface. For the water application study, we firstly used tap water and the results can be found in Fig. 2(b). When the water droplet contact with the polyimide surface, the SHG signal decreased dramatically, indicating that the tribo-induced surface charge diffused due to the water. Finally, the SHG signal reached the lowest state (0.1). It is necessary to point out that the tap water contains many materials that may remain on the surface of the polyimide. Even if the generation of EFI-SHG has good materials selectivity, we cannot confirm that the observed SHG signal is only from the polyimide surface. Therefore, we also prepared distilled pure water (resistivity > 18 MΩcm) and performed the similar experiments, as can be seen in Fig. 2(c). The distilled water also made similar contribution to the charge diffusion and SHG signal returned to the similar lowest level (0.1). Then the Al foil contacted with the polyimide surface again after with the distilled water. The SHG signal increased again, as can be seen in Fig. 2(c). These results proved that the surface charge was indeed removed by liquid water. Finally, the water steam was also applied to the surface of the polyimide film, in order to study if the water molecule can accelerate the diffusion of the surface charges. The result was shown in Fig. 2(d). With the appearance of the water steam, the laser path is blocked due to the strong optical scattering. When the steam is dried out, the SHG signal returns to the similar level before the steam application and no significant changing was observed. The results in the Fig. 2(d) proved that the existence of a thin water layer (steam) on the surface of polyimide film will never enhance the tribo-induced charges to diffuse to the surroundings. More importantly, the evaporation of the water molecule cannot remove the surface charges. Therefore, we conclude that only the motion of the water along the charged surface can “take away” the tribo-induced charges. (We also tried alcohol liquid and similar results have also been found.)

### Multi-contact effect

**Figure 3:**
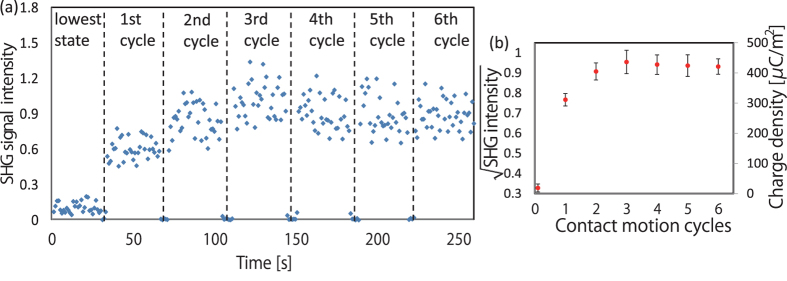
The SHG signal during the multi-contact study of polyimide film, where polyimide film was cleaned by distilled water firstly, in order to reach the lowest surface state. (**b**) The summarized data of SHG intensity and surface charge density after different contact motion cycles.

Since we have already determined the lowest surface potential state by using water application experiments, we can investigate the multi-contact effect on the tribo-charge generation on the interface of polyimide. In this experiment, the polyimide was contacted with Al foil for multiple motion cycles at the same area with constant contact force. The corresponding SHG signal changing after each motion cycle is shown in Fig. 3(a). After the first contact, the average SHG signal increased from 0.1 to about 0.6 and after five motion cycles the SHG signal saturated at about 0.9. It is interesting to find that the SHG signal showed some peak values (SHG intensity 1.0) after 3 or 4 motion cycles and this value decreased slightly before it saturated. We have confirmed this phenomenon by repeating the experiments using different samples. The detailed reason related to the phenomenon still needs further analysis. It is quite possible that the surface morphology will show some small damage during the repeating contact and thus the saturated value of the surface charge density may not be the maximum value. The experimental results with different samples were summarized, as shown in Fig. 3(b). Each point in Fig. 3(b) is calculated from the average value of the SHG intensity within first 10 seconds immediately after the contact. Based on the linear relationship between 
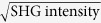
 and the local electric field, the surface charges density was also calculated. The experiments were repeated with four similar samples and the calculated error bar based on standard deviation can also be found in Fig. 3(b).

## Discussion

By continuously monitoring the relaxation of the tribo-induced surface charge, the EFI-SHG measurement in Fig. 1(d) proved that the diffusion of the tribo-induced charges will have multiple relaxation processes and the SHG signal is able to distinguish these relaxations in the time domain. It is necessary to note that for the study of the contact electrification using KPM, we more focus on the time region of more than 1000 seconds, in order to get some stable scanning. Accordingly, the net discharge rates previously observed for macroscopic contact electrified materials usually have a time constant on the order of 10^3^ s^4^,^9^, which is also in good accordance with the second relaxation process (SHG signal from 0.7 to 0.3) we observed. This relaxation process is likely to be the charge relaxation along the surface of the polyimide to the surroundings, which has been studied by the previous research^4^,^9^. Meanwhile, the results in Fig. 1(d) also observed another relaxation process with much smaller time constant (on the order of 10 s), which will be gradually stopped after about 3 minutes. This relaxation process should be the charge relaxation to the air other than through the polyimide film, since the breakdown electric field of air is 3 × 10^6^ V/m while the breakdown electric field of polyimide is 1.3 × 10^8^ V/m^22^. It is also quite possible that the distributed surface energy state is the origin of the different relaxation time constants^14^,^15^. For the application of contact electrification, the dynamic performance of the electrification in the first few minutes or even first few seconds would be important and EFI-SHG system can provide real-time study for this process.

It is also interesting to find that the tribo-excited SHG signal always returns to a base line (0.3) after the second slow relaxation, as shown in Fig. 1(d). The water drop experiment showed that the lowest SHG level is 0.1. Hence, there were some residual tribo-charges on the surface of polyimide film in the so called “steady state” of Fig. 1(d). The induced residual electric field (SHG signal from 0.1 to 0.3) is about 4.6 × 10^6^ V/m (230 V) and charge density is approximately 163 μC/m^2^. These residual charges can usually stay on the surface of polyimide for a quite long time (usually for more than 12 hours). The existence of such residual charges may relate to the presence of some deep energy states on the surface, where the trapping force may balance the diffusion force from the concentration gradient^10^,^14^. Therefore, from the dynamic observation of the EFI-SHG measurements we know that the relaxation of the tribo-induced charges on the surface of polyimide is a quite complex process. So far, we can assume that there will be briefly three processes happened from the saturated surface state. Firstly, within about 180 seconds, a fast relaxation of the charges happened, possibly due to the diffusion into the open air. It is better to study this process by using EFI-SHG because of its good dynamic response characteristics. Secondly, the net discharging along the surface happened with a time constant on the order of 10^3^ s. After this relaxation, the SHG signal returned to 0.3 and showed very slow decrease after that. The SHG signal at this level is caused by the residual charges on the surface, which will experience an extremely long relaxation with a time constant on the order of 10^4^ s (the third relaxation). This multi relaxation process of the tribo-charges has been confirmed by repeating the experiments with different samples.

The water applications in Fig. 2(a–d) designed several special conditions to exhibit the flexibility of EFI-SHG technique. Firstly, we demonstrated that wind from the gas nozzle cannot enhance the diffusion of the surface charges. However, the flowing of the liquid on the charged surface returns the surface potential to the lowest level (see Fig. 2(b)). This phenomenon itself is a well-known experimental method. Nevertheless, the underlying mechanism is worth studying. The steam experiment in the Fig. 2(d) proved that the appearance and the evaporation of a thin water layer also cannot change the surface charge density and the distilled water case also ruled out the possibility of ion neutralization. Therefore, we can conclude that it is the motion of the water along the charged surface can help the tribo-induced charges to diffuse. Namely, the tribo contact between the polyimide and the liquid enhanced the charge diffusion. The good fluidity and permeability of water and alcohol can guarantee the surface return to the lowest potential state. With the help of water application, we can investigate the multi-contact effect on the surface of polyimide, as shown in Fig. 3(a). From the results in Fig. 3(b), we know that the maximum surface charge density is about 440 μC/m^2^ (average value). It is important to note that a latest research based on a triboelectric nano generator using liquid electrode has reported a maximum charge density of 430 μC/m^2^ on the surface of tribo-excited Kapton film, which is in very good agreement with our measurement^23^. The experimental error may come from the errors in electrostatics, such as the discharging in the air or the variation of the surface conditions. Moreover, the laser signal has some fluctuation, which also bring some experimental error to the results.

A comparison of traditional KPM and EFI-SHG is listed in Table 1. Both of these two methods can be applied to study the surface potential distribution and many other electrical properties of the targeted materials. The KPM is to apply electrical signal to balance the electrostatic force generated from the charged surface. The obtained signals from the KPM can be rather stable and have good resolution. However, the moving probe in the KPM measurement may disturb the original electrostatic field. Meanwhile, the probe need to work in the rather strict environment (better in the vacuum) otherwise the electromagnetic interference or some other problem may ruin the measurement. On the contrary, the EFI-SHG is depending on the optical laser signal. It is easy to clear all of the influence signals by using optical filters, since the SHG signal always has fixed laser wavelength. Accordingly, the experimental conditions can be liberated and we can test the adaptability of contact electrification process to some special condition (wind, steam and so on). The resolution of SHG is limited by the wavelength of the probing laser, which is ~1 μm. The data in Fig. 1(b) showed signal fluctuation, of course, the signal stability can be improved by the use of advanced laser such as femto-second laser source^13^. Furthermore, the EFI-SHG can provide continuous real-time observation of the charge relaxation on the surface, which can help us to get some deep understanding about the dynamic performance of the contact electrification. Finally, the EFI-SHG system has promising material selectivity. The different materials respond at different laser wavelength (due to resonant enhancement of optical transition). It is possible for us to rule out the influence from the dust or other surface contaminants, such that we did in the water application’s case. Furthermore, according to the recent research, the contact electrification between two dielectric materials led to a surface with a random “mosaic” of oppositely charged regions of nanoscopic dimensions^4^. It is also possible that we can use different laser wavelengths to scan different materials colony on the same surface. Generally speaking, the defect of the KPM technique is the flexibility of the experimental condition, while the EFI-SHG technique needs a clear physical understanding to analyze the data. It is quite possible that the two techniques can be complementary to each other. The EFI-SHG technique is a new arriver in the field of contact electrification and therefore this technique could have many application prospects in conjunction with the conventional studies and conventional experimental methods. Meanwhile, the real-time observation using EFI-SHG under very flexible experimental condition can also be the technical support for the tribo based energy device, for example, TENG and so on.

In conclusion, by applying the EFI-SHG measurements to the polyimide film, we systematically studied the contact electrification process happen between Al foil and polyimide film. The real-time observation from the EFI-SHG visualized a fast relaxation of tribo-induced charges within 3 min after the tribo-excitation and two slower relaxation processes with the time constants of 10^3^ s and 10^4^ s, respectively. We also designed several special experimental conditions (wind, water and steam) for studying the adaptability of contact electrification process. The results proved that only the motion of the water can remove the tribo-charges on the surface, while the appearance of water steam cannot change the charge diffusion process. The EFI-SHG system is a new approach to study the contact electrification process. Accordingly, we carefully analyzed the advantages and the defects of this method in comparison with the conventional KPM method. The experimental flexibility and material selectivity of the EFI-SHG system can allow many potential applications of this technique in the study field of contact electrification and in the development of electrification based energy devices.

## Methods

### Experimental set up of the EFI-SHG system

**Figure 4:**
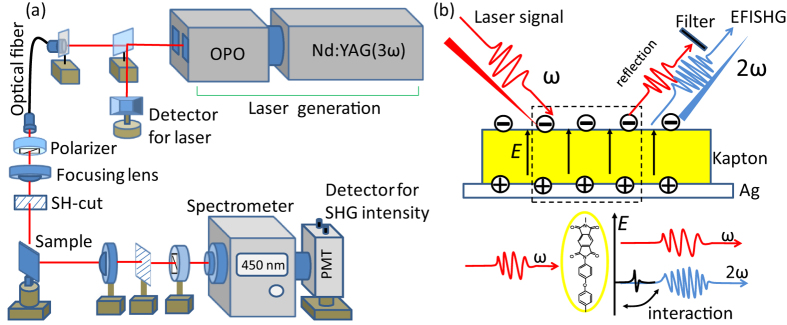
(**a**) Experimental set up of EFI-SHG system (**b**) the sketch of the generation of EFI-SHG signal. All the figures were drawn by X. C.

Figure 4(a) portrays the experimental arrangement used for the EFI-SHG measurements and the photograph of the whole system can be seen in the supplementary info (Fig. S4). A pulsed laser was used as a probing light (repetition rate 10 Hz, average power 1 mW, duration 4 ns), which was generated from an optical parametric oscillator pumped with the third-harmonic light of *Q*-switched Nd:YAG laser. A *p*-polarized pulsed laser beam was focused onto the sample surface at an incident angle of 45°. We inserted two SH-cut filters to remove influence light signal, optical filters and a spectrometer was set in the path of the reflected laser, in order to allow only the second harmonic signals to pass through. The EFI-SHG light generated from the sample was finally detected using the photomultiplier tube (PMT) at the end of the spectrometer, and its intensity was recorded with a digital multimeter. It is important to note that the size of the laser spot is about 0.8 mm^2^ (see Fig. S5(a)) and the EFI-SHG system in Fig. 4(a) is recording the average SHG intensity in this laser region. In order to further develop the resolution in the 2 dimensional (2-D) region, we need to use photographing technique to catch the SHG image as we did in our previous research^12^. The 2-D mapping of the polyimide film without and with SHG generation can be seen in Fig. S5(b) and (c). In Fig. S5(b) and (c), the light intensity was caught by the high-sensitivity cooled CCD, where a simple filter was also applied to remove the fundamental laser and only observe the SHG signal. This measurement can allow us to obtain detailed intensity distribution within the laser spot, where the highest resolution is about 20 μm. In current study, we used the EFI-SHG shown in Fig. 4(a) and only showed the average intensity of the SHG signal from the whole laser spot, as can be seen in Figs. 1, 2, 3.

### EFI-SHG generation

By laser irradiation, EFI-SHG signal is generated from polyimide film due to the coupling of electrons in molecules and electro-magnetic waves *E*(ω) in the presence of electrostatic local electric field *E*(0) (The “0” means that it is static field), as can be seen in Fig. 4(b). The SHG response from polyimide film should originate mostly from the first few nanometers region near the surface of dielectric film, since the signal from the bulk region may extinct due to the optical absorption^24^. The EFI-SHG intensity is given as^24^,^25^:


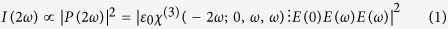


where *P*(2ω) is the nonlinear polarization induced in the polyimide film, ε_0_ is the vacuum permittivity, and χ^(3)^ is the third order nonlinear susceptibility tensor. *E*(0) is the electrostatic local field in the organic layers (we define as that positive electric field *E*(0) points from the Ag electrode to the top of polyimide film), and *E*(ω) is the electric field of incident laser beam. Equation (1) indicates that the square-root of the SHG signal intensity is in proportion to the electric field *E*(0) in the polyimide film. Here, *E*(0) is given by *E* = *Q*_*m*_/*ε*_*0*_*ε*, where charges *Q*_*m*_ is the charge on top and bottom surface of the polyimide and *ε* is the relative permittivity of polyimide. The susceptibility tensor χ^(3)^ in eq. (1) is a material dependent parameter and is a function of the optical frequency ω. The difference in χ^(3)^ among materials allows us to selectively probe electric field only in the polyimide film with an appropriate laser beam wavelength^16^. In the present study, we used the laser beam at a wavelength of 900 nm and recorded the generated SHG at a wavelength of 450 nm to selectively measure the electric field in polyimide film. We found that this wavelength is the only optimized wavelength for polyimide. The absorption spectrum of the polyimide was shown in Fig. S6. The water molecules at surface is possible source of SHG signal^16^,^17^, while these are negligibly small compared with the SHG from the polyimide. Hence, it is possible to apply water to the surface of the polyimide in the SHG experiments if it will not block the laser path. This also can be the demonstration of the material selectivity of the EFI-SHG technique for characterizing the contact electrification process.

## Additional Information

**How to cite this article**: Chen, X. *et al.* Direct probing of contact electrification by using optical second harmonic generation technique. *Sci. Rep.* **5**, 13019; doi: 10.1038/srep13019 (2015).

## Supplementary Material

Supplementary Information

## Figures and Tables

**Table 1:** The comparison between KPM and EFI-SHG.

	**Kelvin probe microscopy (KPM)**	**EFI-SHG**
Measurement type	Electrical, contact mode	Optical, non-contact mode
Applied signal	Voltage signal to balance the electrostatic force	Laser signal to excite the sample
Obtained signal	Stable voltage signal with high spatial resolution	Optical signal that is proportional to the strength of electric field
Potential measurement	Surface potential distribution, surface charge density and so on.	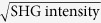 ∝ field strength, Potential study is also possible.
Experimental conditions	Strict condition, sometimes in the vacuum. The probe itself may disturb the surface electrostatic field	Very flexible and no electromagnetic interference. Sample can be with wind, steam and many other conditions
Real time monitoring	The real-time study is partially possible.	Laser signal can continuously detect the sample during the whole experiments.
Materials selectivity	No selectivity, surface contaminants and particles can be the problem.	Distinguishable, different materials react to different laser wavelength.
Defects	Only suitable for the limited experimental conditions	Analyzing SHG signal need a clear physical understanding
