# Supplementary material for: Direct probing of contact electrification by using optical second harmonic generation technique
Source: Sci Rep. 2015 Aug 14;5:13019. doi: 10.1038/srep13019 (PMC4536524; doi:10.1038/srep13019)
Supplement: Supplementary Information [file srep13019-s1.pdf]

Supplementary Information

Direct probing of contact electrification by using optical second harmonics generation technique

Xiangyu Chen[a], Dai Taguchi[b], Takaaki Manaka[b], Mitsumasa Iwamoto[b]* and Zhong Lin Wang[ac]*

a. *Beijing Institute of Nanoenergy and Nanosystems, Chinese Academy of Sciences, Beijing 100083, China,*

b. *Department of Physical Electronics, Tokyo Institute of Technology, 2-12-1 S3-33 O-okayama, Meguro-ku Tokyo 152-8552 Japan*

c. *School of Materials Science and Engineering, Georgia Institute of Technology, Atlanta, Georgia 30332-0245, USA.*

E-mail: *iwamoto@pe.titech.ac.jp (M. I.), zlwang@gatech.edu (Z. L. W.)*

1. Information for discussion

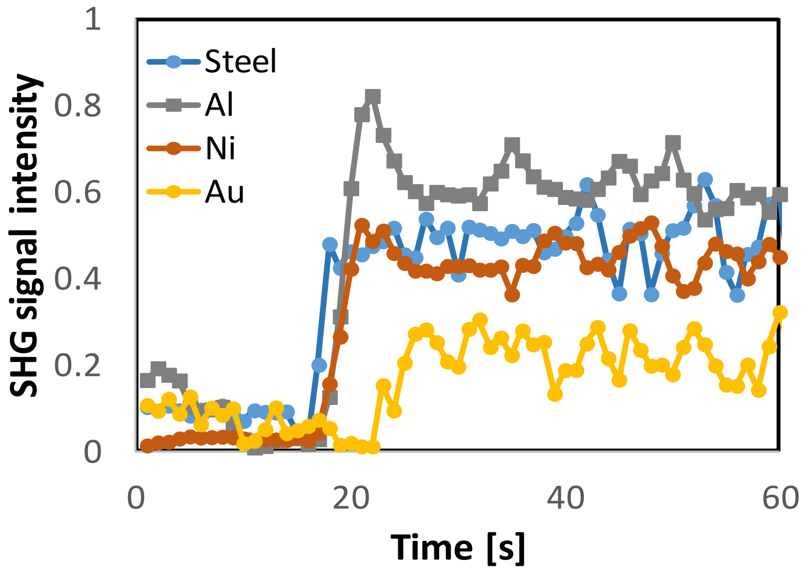

Figure S1 The SHG signal SHG generated from the polyimide film contacted with different metal. All the figure were drawn by X.C.

As shown in Fig. S1, the changes of the SHG signal with Al, Ni and Steel were almost the same and Al show the highest electrification ability. The Au foil generated much lower SHG signal, which may be due to the soft Au foil we used that can not sustain enough contact force. Here, the polyimide film was cleaned by Alcohol before each contact in order to normalize the experiments. Finally, we use Al in our later experiments. All the figure were drawn by X.C.

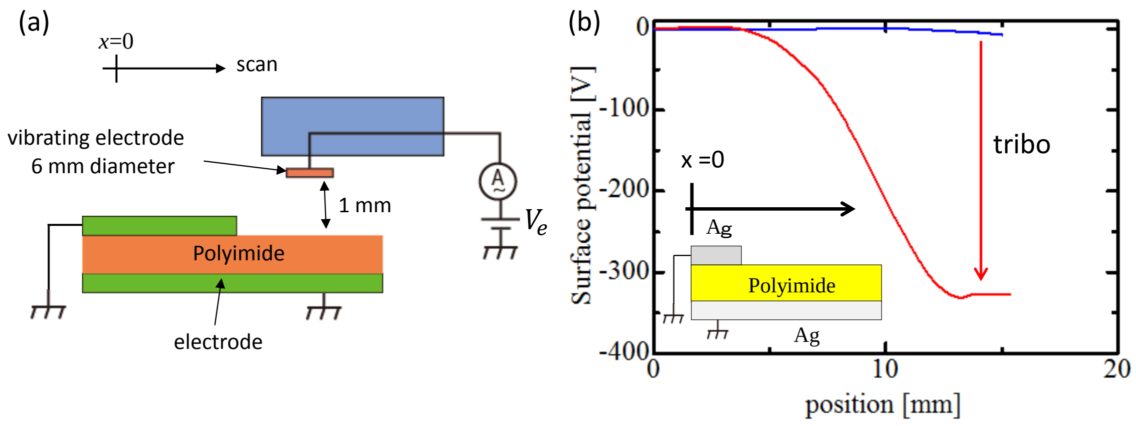

Figure S2 (a) the set-up for Kelvin probe measurement (b) the obtained surface potential during the scan of the polyimide surface, which was contacted by the Al foil after four motion cycles.

The set-up of the Kelvin probe measurement was shown in Fig. S2(a). Here the surface potential of the polyimide (Kapton) film was measured using vibrating probe. This measurement is the typical Kelvin probe measurement, where the voltage was applied on the probing electrode to balance the electrostatic force caused by the charged surface. The whole measurements were conducted in the vacuum. The results can be seen in Fig. S2(b). The surface potential is about -340 V, which can be used to compare with the estimation from the SHG signal.

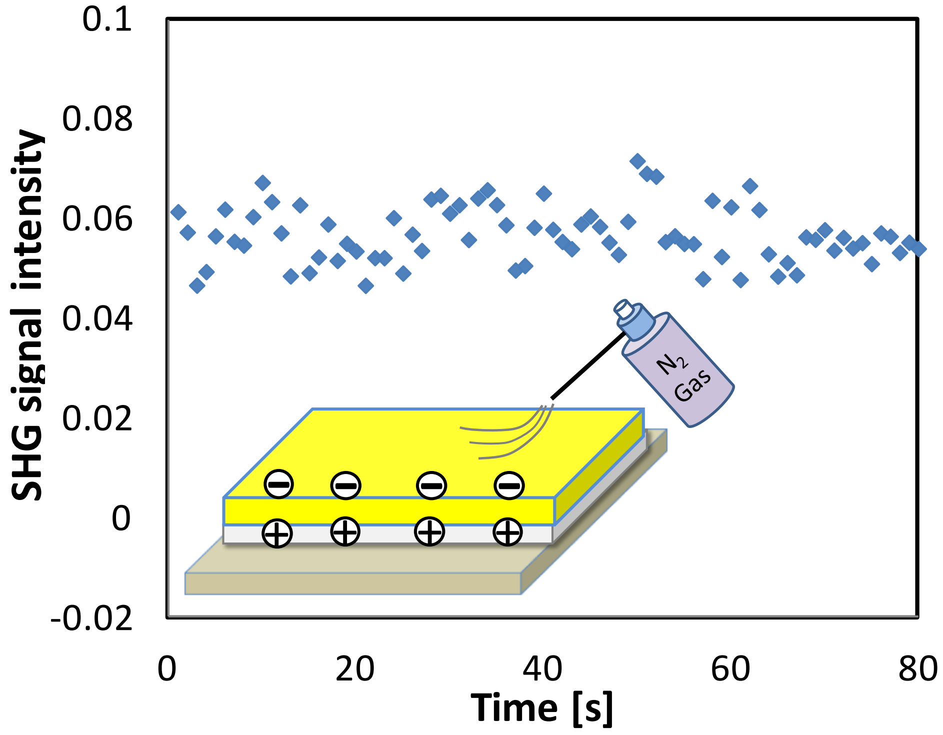

Figure S3 The SHG signal from the charged polyimide film under the continuous wind from the gas duster, where SHG signal show no significant changing. All the figure were drawn by X.C.

2. Information for experimental methods

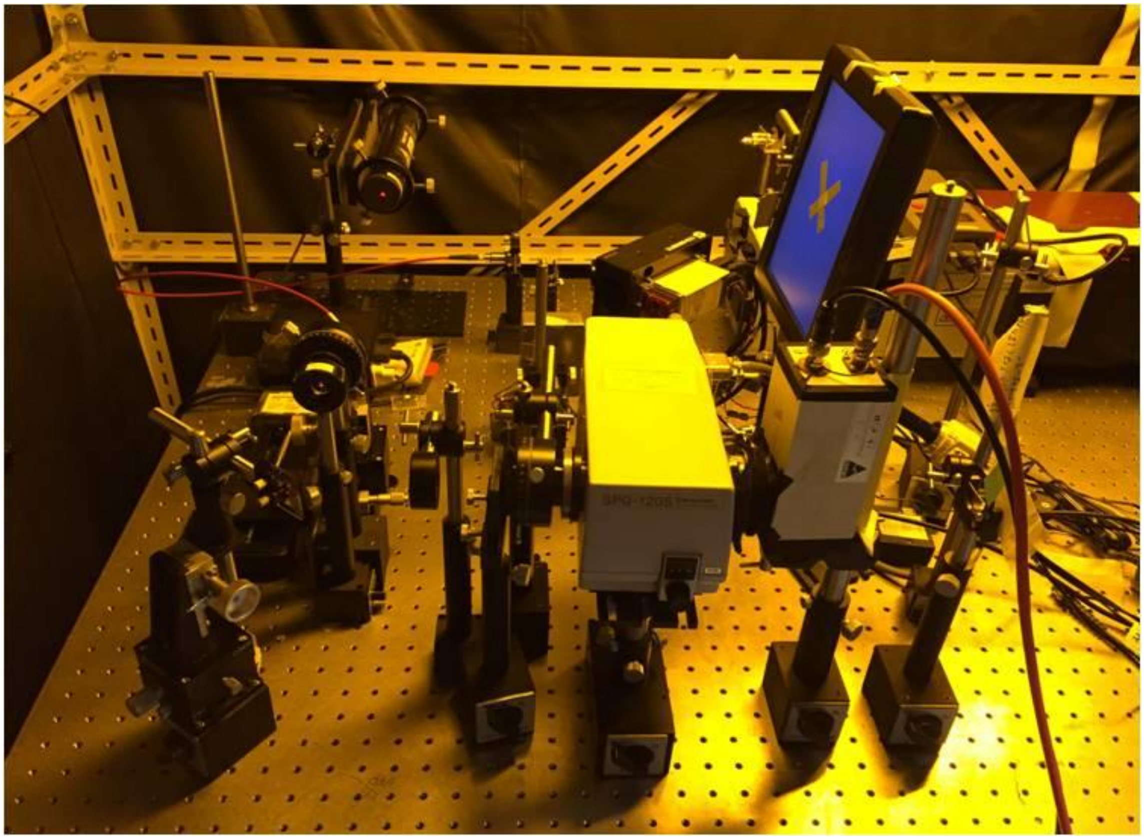

Figure S4 The photograph of the EFI-SHG system.

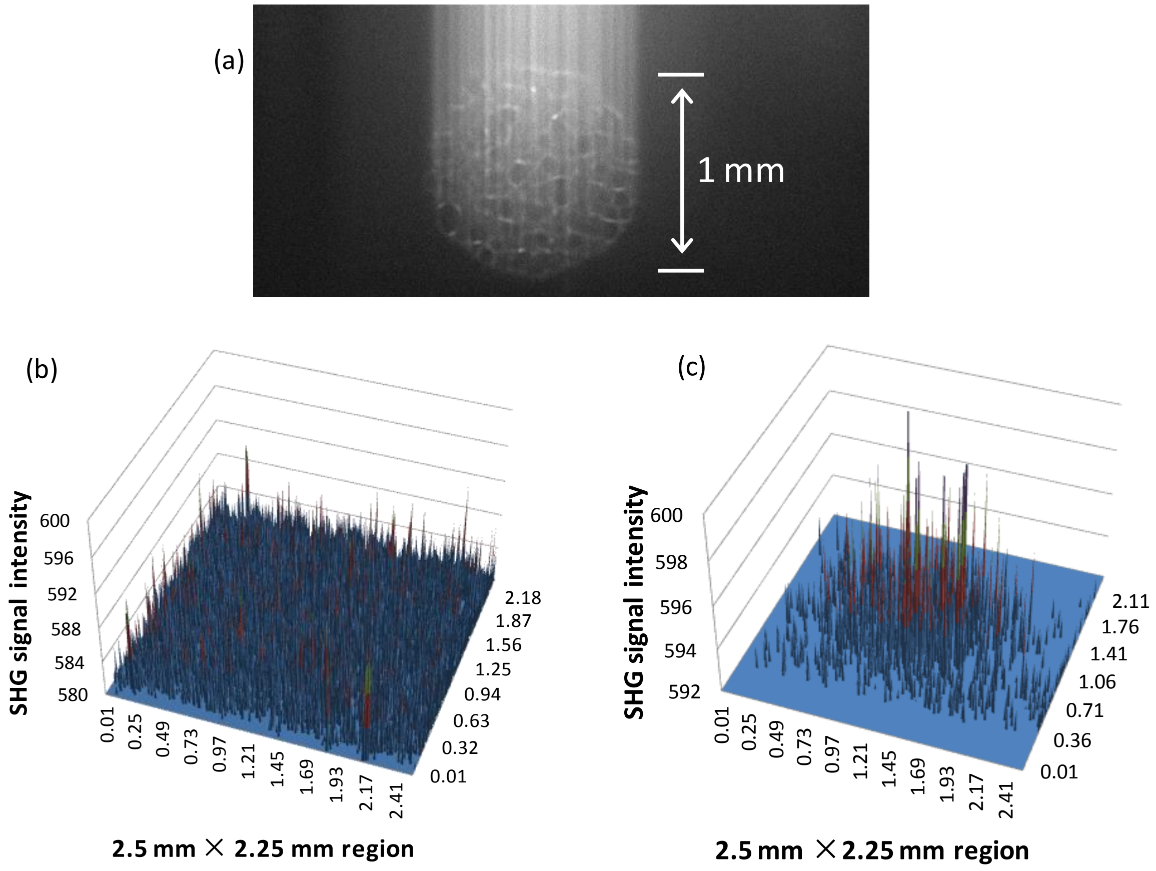

Figure S5 (a) the image from the CCD camera to illustrate the size of the laser spot (b) the SHG intensity of the polyimide film when the laser signal is off, where the exhibiting region is a square of 2.5 mm× 2.25 mm. (c) the SHG intensity from the polyimide film under the laser excitation, where the regions is still 2.5 mm× 2.25 mm.

Figure S3(a) is the image of the laser spot. In Fig. S3(b) and S3(c), the light intensity was caught by the high-sensitivity cooled CCD, where a simple filter was also applied to remove the fundamental laser and only observe the SHG signal. It is important to know that the SHG signal is much smaller than the fundamental laser signal. With the generation of SHG, the light intensity in Fig. S3(c) was increased significantly in comparison with Fig. S3(b), where the laser signal is off. This measurement can allow us to obtain detailed intensity distribution within the laser spot, where the highest resolution is about 20 μm.

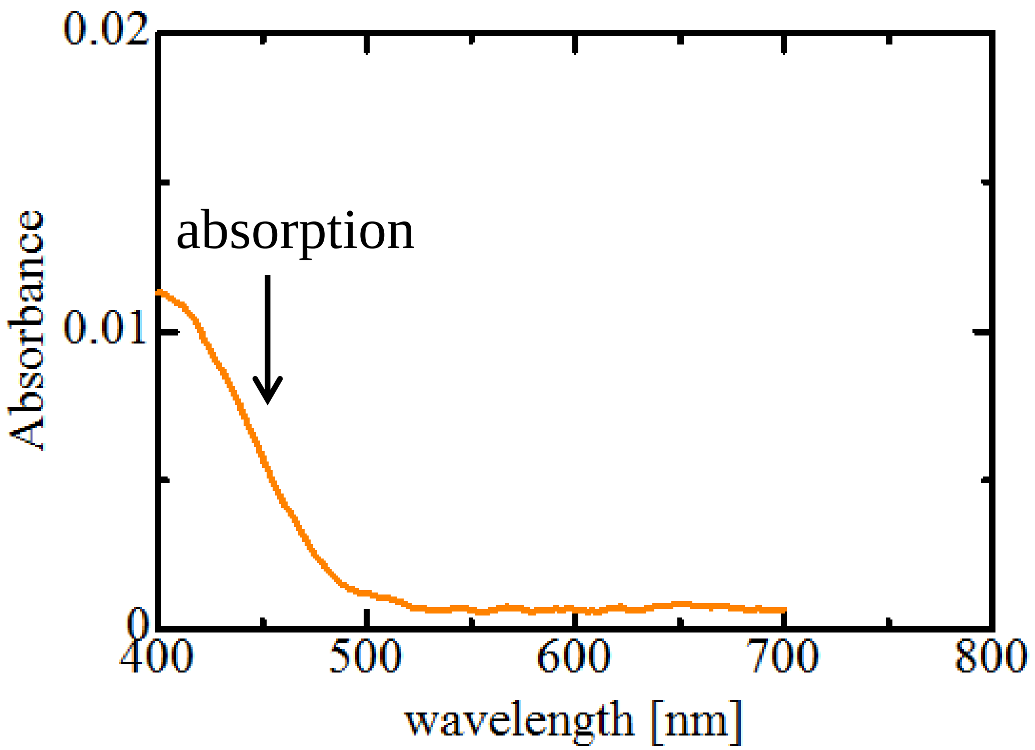

Figure. S6 The absorption spectra from the polyimide film.

Here, the absorption spectra was shown in Fig. S6. The absorption happened in the wavelength smaller than 500 nm. Hence, we will start to search the optimized laser wavelength in this region. By repeating the experiments, we found that the best laser wavelength to couple with polyimide (Kapton) is about 900 nm and SHG signal is 450 nm.
